# Give until It Hurts: An Exploratory Analysis of Mental Health Workers’ Wellness

**DOI:** 10.3390/ijerph20206925

**Published:** 2023-10-14

**Authors:** Marin G. Olson, Karly M. Pyles, Danielle Kristen Nadorff

**Affiliations:** Department of Psychology, Mississippi State University, Starkville, MS 39762, USA; mo606@msstate.edu (M.G.O.); kmp690@msstate.edu (K.M.P.)

**Keywords:** well-being, self-care, burnout, anxiety, depression, mental health workers

## Abstract

Background: The well-being of mental health professionals has been of growing concern due to the increasing rates of reported stress and burnout. Previous research suggests that the quality of life for mental health workers (MHWs) is at an increased risk due to clinical load, salary concerns, and lack of time for self-care activities outside of work. There is a lack of research regarding the wellness of MHWs (i.e., psychology graduate students, academic faculty, psychiatrists, and mental health counselors) and its relation to the workplace environment. This study examined job-related factors that impacted participants’ social, emotional, and professional well-being. Methods: Participants were recruited via professional organization listservs and answered questions about their psychological health (Generalized Anxiety Disorder-7 and Patient Health Questionnaire-9), support systems (Multidimensional Scale of Perceived Social Support), and three qualitative questions about what they enjoy or find challenging about their work and any barriers to engaging in self-care activities. Results: An inductive qualitative analysis of the qualitative questions and descriptive statistics are presented to provide context for their qualitative responses. Conclusions: Institutions can better support their workers by creating outlets to teach their staff self-care strategies and practice these strategies at work.

## 1. Introduction

“You cannot serve from an empty vessel.”—Eleanor Brownn

The mental health field draws in countless individuals who aim to help others. In the United States, approximately 2.2 million people work in the mental health field, encapsulating anyone who works as a social and human service assistant to a psychiatrist [1]. Though the mental health field may appear robust, concerns about recruiting and retaining workers to maintain and improve patient care quality persist [2,3]. In tandem with this concern, there is an ever-increasing demand for mental health services in the United States that only escalated in the wake of the COVID-19 pandemic [4,5,6], and this creates a situation where mental health workers (MHWs) are hard pressed to meet this demand. In addition to those in the mental health field who work more directly with clients, there are those who serve as educators to future clinicians and researchers who are also impacted by the stressors associated with working in the mental health field [7]. With already emotionally and mentally strenuous occupations, the added pressure of the population’s increasing need for mental health care places MHWs at an increased risk for burnout, depression, and anxiety [3,8]. 

Burnout syndrome is best described as the result of prolonged emotional exhaustion, stress, professional inefficacy, cynicism, and dissatisfaction with one’s work [9,10,11]. Burnout syndrome has been shown to be a distinct and valid entity in its own right and is often associated with increased symptoms of depression and anxiety in workers [7,10,11]. Burnout is also associated with increased rates of employee turnover and employee healthcare costs [2], and more troublingly, MHW burnout has been proven to negatively affect patient outcomes [12]. In essence, MHW’s well-being must be a priority for mental health institutions and organizations for their operations and society at large to run efficiently [13]. 

A theory that can account for how burnout syndrome manifests through one’s occupation is the Job Demands-Resources Model, which “attributes employee well-being to the characteristics of work environments” [14]. Workplace factors that contribute to the prevalence of burnout in MHWs include high workloads or caseloads, lack of community (i.e., having few relationships at work, inadequate supervision, poor role clarity, and few reinforcements for quality work), having a sense of unfairness, and less autonomy [3,10,15,16]. In contrast, elements that may prevent or reduce burnout are social support, having regular access to supportive supervisors, workplace interventions to help reduce stress and increase cohesion between workers, and practicing regular self-care [15,17,18,19]. 

In the wake of the COVID-19 pandemic, an area that remains to be empirically examined within MHWs is self-care practices. Self-care activities have historically been defined as tending to one’s bodily needs (e.g., using the bathroom, eating, drinking water, resting at appropriate intervals; [20], engaging in behaviors that maintain a person’s overall well-being and decreasing one’s emotional distress) [21,22]. As the name implies, self-care has historically been viewed as an individual’s responsibility exclusive unto themselves [20,21,22]. Recently, the literature has extended the onus of workers’ self-care practices to the institutions where they work, as institutions create environments that may not be conducive to workers meeting their own needs due to demanding schedules, high cognitive load, and significant emotional burden from clients [16,20,21,22]. Some institutions have had success with providing outlets for their employees to learn about self-care and engage in wellness activities as a means of supporting their workers [21,22,23,24,25]. Generally, the ideal situation would be for workers to practice self-care with the support of their employers; however, it remains to be seen if this dynamic is the experience of MHWs today and how this contributes to burnout. 

Research surrounding burnout for MHWs began gaining traction in the late 1970s, focusing on staff members at inpatient facilities [26], and has slowly evolved to include a wider range of workers and use more rigorous methods of study [27]. Since then, research progressed to expand what is known about how burnout comes about and what factors exacerbate the syndrome [28,29,30]. As the trail to burnout became clearer, studies about burnout interventions became more popular in the literature [31]. Over the course of the last 50 years of burnout research, numerous narrative and literature reviews and meta-analyses have been conducted on the subject with a re-emphasis on preventing and treating burnout [2,11,15,27]. Although these reviews and meta-analyses do contribute to the field, these studies are limited by the fact that their findings are based on older data. With the increase in mental health awareness within the United States and the worldwide changes that came in the wake of the COVID-19 pandemic, it is possible that burnout rates in MHWs may have changed, and these changes would be best observed with current data. 

Throughout MHW burnout research, one of the most consistently used methods is surveying MHWs with self-report measures [3,10,13,15,32]. Due to the literature’s emphasis on finding connections between social support, depression, and anxiety and understanding how they impact burnout, many studies have used the following validated measures in varying combinations: Multidimensional Scale of Perceived Social Support [33], Generalized Anxiety Disorder Screener [34], and Patient Health Questionnaire [17,35]. 

### Current Study

The wellness of clinical psychologists and mental health professionals has been of growing concern due to the increasing rates of reported stress and burnout [3,7,8]. Previous research suggests that the quality of life for mental health professionals is at an increased risk due to high clinical loads, salary concerns, and lack of time for self-care activities outside of work [3]. There is a lack of research regarding the wellness of mental health practitioners (i.e., psychology graduate students, academic faculty, psychiatrists, and mental health counselors) and its relation to the workplace environment. The purpose of this exploratory mixed-methods study is to determine which and to what extent job-related factors impact participants’ social, emotional, and professional well-being. With this aim in mind, the following questions are considered:

(1) What is the current emotional state of mental health workers?
(2) What elements of mental health workers’ working conditions add to or detract from their mental health?

## 2. Materials and Methods

### 2.1. Participants

Participants were recruited using convenience sampling methods via various professional psychology listservs to complete a cross-sectional web-based survey from April–June 2023. Sixty-five participants opened the survey and completed up to the demographic questions, and 60 mental health workers (73% identified as female, 92% identified as White, and the average age was 44) data was included in the analysis of the study’s results (i.e., some participants missed responding to some questions or discontinued responding later in the survey; see Table 1 for demographic information).

### 2.2. Materials

The measures included the Patient Health Questionnaire (PHQ-9), Generalized Anxiety Disorder Screener (GAD-7), Multidimensional Scale of Perceived Social Support (MSPSS), and three qualitative questions. 

#### 2.2.1. Patient Health Questionnaire-9 (PHQ-9)

The Patient Health Questionnaire-9 (PHQ-9) is a nine-item self-report measure that was designed to screen and gauge an individual’s depressive symptoms [35]. Responses are recorded on a four-point Likert scale that ranges from 0 to 3, where scores may range from 0–27, and higher scores indicate the presence of more depressive symptoms [35,36]. The PHQ-9′s psychometric properties indicate that it has decent discriminant and convergent validity, in addition to good internal consistency with Cronbach’s α = 0.74 [35,37,38]. 

#### 2.2.2. Generalized Anxiety Disorder-7 (GAD-7)

The Generalized Anxiety Disorder Screener (GAD-7) is a seven-item self-report measure that was designed to screen and measure the presence and severity of generalized anxiety disorder symptoms in primary care settings [34,39]. Responses are recorded via a four-point Likert scale, and scores range from 0–21, with higher scores indicating more severe anxiety symptoms [34]. The GAD-7’s psychometric properties indicate that it has acceptable construct, discriminant, and content validity, in addition to ideal internal consistency Cronbach’s α = 0.88 [34,39,40]. 

#### 2.2.3. Multidimensional Scale of Perceived Social Support (MSPSS)

The Multidimensional Scale of Perceived Social Support (MSPSS) was designed to measure an individual’s perceived social support through 12 Likert scale questions, where scores can range from 1–7, and higher scores indicate higher levels of perceived social support [17,33,41]. The MSPSS has been shown to be a valid and reliable measure of social support (α = 0.91), with each of the three subscales (support from friends, family, and significant others) having a Cronbach alpha of 0.90 or higher [41]. 

### 2.3. Procedures

The qualitative data were analyzed via an inductive qualitative analysis [42,43], while the quantitative data were analyzed with descriptive statistics and correlations. 

The authors completed an independent parallel coding of the data, and the resulting subthemes of this preliminary analysis were condensed into the target themes [43]. These themes were then compiled into a codebook for the two research assistants (both had a psychology background: one was in their senior year of their bachelor’s degree, and the other was a second-year clinical psychology doctoral student) to use for identifying themes within the participant responses. An interrater reliability score was developed for each theme (see Table 2, Table 3 and Table 4). Participants were sorted into one of four categories based on how they characterized their job description: mental health workers (e.g., therapists, mental health nurses, social workers, etc.), graduate students, clinical psychologists, and researchers. These categories were used to calculate frequency counts for each theme and proportions for each group so that comparisons may be drawn by position (see Table 5).

## 3. Results

### 3.1. Qualitative Results

There were three open-answer questions that participants responded to:

1. What are two things you wish were different about your job?
2. What are two things you love about your job?
3. What prevents you from engaging in self-care activities?

Two themes were identified for question one—wishing for more resources and requesting more support (see Table 2 and Table 5). The “need for more resources" theme refers to participants requesting more time to complete tasks, increased compensation, thorough training and supervision, and access to community resources to offer referrals for their clientele. The “need for more support” theme was developed based on several responses that workers are seeking more manageable workloads, sensitive managers, improving interpersonal relationships between workers, and policies that are more ergonomic to employee needs. Interestingly, the two highest reporting groups for the “resources” theme were graduate students or clinical psychologists, followed by mental health workers. For the “support” theme, the highest reporters were those who identified as clinical psychologists, followed by mental health workers and graduate students. 

There were four themes identified for question two: (1) enjoying the work with clients, (2) relationships with colleagues, (3) worker independence, (4) upholding values, and (5) mental challenge and advancement (see Table 3 and Table 5). Theme 1 referred to anything redeeming about working with individuals receiving mental health services. Theme 2 referred to enjoying working with any teams or individuals the mental health worker interacts with regularly. Theme 3 captured mental health workers’ acknowledgment of having any amount of autonomy in any aspect of their work. Theme 4 was developed based on statements that discussed how mental health workers enjoy feeling like they are making a difference or positive impact through their occupation. The fifth theme is how some mental health workers described their work as a positive challenge, augments their skillset, or increases their knowledge base about something novel.

The greatest group proportion for Theme 1 (i.e., “enjoying work with clients”) came from graduate students and researchers (*n* = 8; 47%), followed by clinical psychologists (*n* = 10; 50%) and mental health workers (*n* = 6; 29%). Graduate students and researchers were also the most frequent reporters of their relationships with colleagues being a source of joy within their work (*n* = 8; 47%), and clinical psychologists were the second highest reporters (*n* =7; 35%) for this theme. The most frequent reporters for the independence theme were the clinical psychologists (*n* = 7; 35%); however, the participant group with the greatest proportion of reporters for this theme was researchers (*n* =2; 100%). For the “upholding personal values theme”, the group with the greatest frequency and proportion of participants was the mental health workers (*n* = 9; 43%), and the second greatest group was graduate students and researchers (*n* = 5; 29%). For the mental challenge and advancement theme, 18% of graduate students and researchers (*n* = 5) and 20% of clinical psychologists (*n* = 4) made remarks that aligned with this theme, followed by mental health workers (*n* = 2; 10%). 

Three themes were detected for question three: (1) time, (2) internal factors, and (3) external factors (see Table 4 and Table 5). The “time” theme was created based on numerous mentions of not having enough time to engage in self-care activities after work and meet basic needs (e.g., eating and sleeping). The “internal factors” theme encompasses any feelings of burnout (e.g., stress, overwhelmed, depression, tired) and other factors that would likely only impact the individual directly. Theme 3 contrasts with the “internal factors” theme in that it refers to aspects of a participant’s work or life that are beyond their control and impact their ability to engage in self-care activities, such as workload, commute, access to resources, familial obligations, and staffing at work. 

The group with the highest frequency for the “time” theme was mental health workers (*n* = 10; 48%). Graduate students and researchers were the second-highest reporters for this theme (*n* = 9; 53%). This trend occurred for the “external factors” theme, where mental health workers had the highest frequency and proportion of responses (*n* = 6; 29%), and the graduate student and researchers’ group had the second highest frequency count and proportion for this theme (*n* = 5; 29%). For the “internal factors” theme, graduate students and researchers had the highest frequency and proportion compared to the other three groups (*n* = 10; 59%) and were followed by clinical psychologists (*n* = 7; 35%). 

### 3.2. Quantitative Results

Of the 60 participants who responded, 55 participants attempted and completed each of the three quantitative measures: the Generalized Anxiety Disorder 7-item questionnaire (GAD-7), Patient Health Questionnaire-9 (PHQ-9), and Multidimensional Scale of Perceived Social Support (MSPSS). The 5 participants who did not attempt the three measures were not included in the analysis. Descriptive statistics for each measure were calculated using IBM SPSS Statistics Version 22 (see Table 6). The participants’ average score on the GAD-7 was 12.4, and the mode was 10. Individuals with similar scores (10–14) report moderate anxiety symptoms [34]. Participants’ scores average score on the PHQ-9 was 14.05, and the mode was 10 (see Table 6). Individuals with similar scores (10–14) report moderate depressive symptoms and would likely benefit from mental health support, such as pharmacotherapy or counseling [36]. The other measure that participants completed was the MSPSS, where the average total score was 5.8, and the mode was 7 (see Table 6). Individuals with similar scores (5.1–7) report high levels of social support [33]. 

To explore potential relations between perceived social support (MSPSS), anxiety (GAD-7), and depressive symptoms (PHQ-9) reported by participants, several Pearson correlations were conducted using IBM SPSS Statistics Version 22 between the overall total scores for each of the GAD-7, PHQ-9, MSPSS, and the subscales of the MSPSS. A significant negative correlation was identified between the MSPSS’s family subscale and depressive symptoms reported on the PHQ-9, *r*(53) = −0.29 *p* = 0.03. This relation may be interpreted as MHWs’ family support being mildly protective against experiencing significant depressive symptoms. Such a relation was not found between any aspect of perceived social support and anxiety. These results indicate that though social support can be found outside of the workplace, it is not a sufficient resource to protect MHWs from symptoms of depression, anxiety, and burnout. 

## 4. Discussion

The current study offers insights into the well-being of mental health workers (MHWs) in a variety of settings and positions (e.g., graduate programs, psychologists, providing direct client care, and research). The results of this study indicate that MHWs are experiencing moderate levels of anxiety and depression on the GAD-7 and PHQ-9. The participants’ qualitative responses about aspects of their work situation that they wish were different included having more resources and support from their organizational leadership. Examples of resources that participants requested were getting more time to complete tasks and time off, higher pay, more training opportunities, and increased access to supervision. Participants’ reports of realistic and helpful resources are congruent with the findings of Williams and colleagues’ who stated that having appropriate access to supervision and reasonable workloads decreases work-related stress and burnout [3]. Participants shared that they would feel more supported by having more autonomy and respect within the workplace, having opportunities to strengthen relationships with colleagues and leaders, and having policies that are more complimentary to their job’s demands. These sentiments are consistent with the findings of McFadden and colleagues’ 2020 study, which indicated that organizational and management support are helpful in preventing and treating burnout symptoms in health service workers [19]. 

In tandem with these appeals, MHWs reported several qualities of their work that bring them joy. Specifically, MHWs reported that they enjoy their work with clients, feeling as though they are working in line with their values, their relationships with colleagues, their independence (although this heavily depended on the position the participant held), and the variety of mental challenges they encountered in their work. An implication for these findings is that they can inform workplace interventions to help support MHWs to decrease their levels of burnout and keep MHWs in touch with the elements of their work that they find most rewarding. 

In addition to these findings, mental health workers reported that they struggled to engage in self-care activities and characterized the barriers to self-care as internal difficulties (e.g., feeling stressed, burned out, overwhelmed), external factors (e.g., workload, commute, lack of access to resources, familial obligations, staffing), and lack of time. Despite this study’s participants reporting on the MSPSS that they have strong support systems and that the existing literature indicates that connecting with their support system can function as self-care to decrease a person’s symptoms of burnout [3,17]; however, this study’s findings indicate that social support is not an adequate protective factor from MHWs experiencing anxiety and depression. 

These findings are consistent with the subject literature in that workplaces, institutions, and employers have a significant impact on their employees [14]. These findings also speak to how self-care is best practiced by individuals with institutional support [3,10,15,24]. 

### Limitations and Future Directions

Although the current study offers valuable insights into the state of affairs for MHWs, there are some limitations and future directions that are worthy of exploration. As this study was exploratory, the sample size was small, and the recruitment was not wholly inclusive in terms of professional identity and racial and ethnic identity. As approximately 88% of United States mental health workers identify as White [44], and 92% of participants identified as White, and psychological professionals are two times more likely to identify as female [45], collecting data from a more representative sample would be a prudent aim for future studies. The researchers sent the survey to several professional organizations that mostly catered to psychologists and psychologists in training. Even though it is believed that this study’s findings may be safely generalized to other branches of the mental health field, gathering a more inclusive sample of MHWs to include more medical staff, social workers, and administrators will offer a greater representation of the field. Recruiting a larger sample in the future will also prevent the necessity of condensing numerous participants into one category (i.e., MHWs), which detracts from representing a more diverse array of mental health professionals and their experiences. Another potential future direction if a larger sample was collected is comparisons could be drawn between participants based on the demographic information they provide. However, a strength of this study was that there was an even distribution of participants from rural and metropolitan areas. 

An additional limitation was that this study collected data at one timepoint instead of collecting data on multiple occasions. Collecting data more than once would have protected against potential over or under-reporting of burnout, anxiety, and depressive symptoms. Another way future studies may control this limitation is to gather data from multiple informants instead of only using self-report measures. Although several studies have used this format of using only self-report measures [3,17,18,20,25], the literature would likely benefit from diversifying its measurement approaches. 

## 5. Conclusions

Mental health workers are reporting that they are experiencing moderate levels of anxiety and depression. Despite receiving support outside of the workplace, MHWs would likely benefit from more resources and support from their institutions. These resources and supports can take the form of institutions creating a work culture where they support employees and train them in practical ways to engage in self-care and create outlets for them to practice these skills. 

## Figures and Tables

**Table 1:** Sociodemographic Characteristics of Participants.

Participant Characteristics	*n*	*%*
Gender	60	
Female	44	73%
Male	13	22%
Gender diverse	3	5%
Racial and Ethnic Identity	60	
Asian American	2	3%
Black	1	2%
White	55	92%
White, European Jewish	1	2%
Hispanic or Latinx	1	2%
Highest educational level	60	
High School or GED	1	2%
Some College	1	2%
Associate Degree	2	4%
Bachelor’s Degree	5	8%
Master’s Degree	12	24%
Doctoral Degree	22	37%
Professional Degree (e.g., PsyD)	17	34%
Licensure Status	60	
Currently Licensed	38	63%
Pursuing Licensure	14	23%
Not Licensed	8	13%
Area of Work	59	
Very Rural	2	3%
Small Town	11	19%
Suburban Area	15	25%
Urban Area	19	32%
Major Metropolitan Area	12	20%

Note. Some participants provided more details than others about their area of work, introducing some variance in the reported N.

**Table 2:** What are two things you wish were different about your job?

Themes	Interrater Reliability Score/% Agreement	Sample Quotes
Need for more resources	88	“More space, more pay.”
		“I wish we were paid more and I wish we had more training in empirically supported treatments prior to seeing clients.”
		“...more overall resources available for when patients need a different type of care.”
Need for more support	68	“connection [with] more colleagues; less paperwork.”
		“Love my job, more interpersonal opportunities.”
		“Work/life balance...”
		“More inclusive language used among providers...”

**Table 3:** What are two things you love about your job?

Themes	Interrater Reliability Score/% Agreement	Sample Quotes
Relationships with colleagues	100	“Experienced coworkers and excellent supervision.”
		“Helpful, thoughtful, and caring coworkers and supervisors.”
Enjoying work with clients	93	“Working with patients in general brings me so much joy.”
		“[D]irect client contact, participating in clients’ growth and healing.”
Worker independence	86	“Being my own boss/autonomy...”
		“I like the flexibility of remote work.”
Upholding personal values	74	“It provides an opportunity to help people in need...”
		“Making a difference.”
Mental challenge and advancement	69	“I get to continuously learn.”
		“...intellectual stimulation via interesting and challenging cases.”

**Table 4:** What prevents you from engaging in self-care activities?

Themes	Interrater Reliability Score/% Agreement	Sample Quotes
Internal factors	89	“Feeling overwhelmed, stressed, and burnt out.”
		“Prioritizing sleep (which is also self-care, but I would like more time to be social and work out).
External factors	89	“[L]onger hours/prioritizing work over self-care.”
		“Balancing work stress and then going home.”
		“Understaffing.”
		“Inversing (mandatory overtime), life situations.”
Time	84	“Lack of time...”

**Table 5:** Frequency Count for Qualitative Themes by Job Title.

Questions	Job Titles					
Themes	Mental Health Workers *		Graduate Students and Researchers		Clinical Psychologists	
	*n* = 21		*n* = 17		*n* = 20	
	*f*	%	*f*	%	*f*	%
What are two things you wish were different about your job?						
Need for more resources	4	19%	12	71%	11	55%
Need for more support	9	43%	11	65%	13	65%
What are two things you love about your job?						
Enjoying work with clients	6	29%	8	47%	10	50%
Relationships with colleagues	7	33%	8	47%	7	35%
Worker independence	3	14%	3	18%	7	35%
Upholding personal values	9	43%	5	29%	2	10%
Mental challenge and advancement	2	10%	3	18%	4	20%
What prevents you from engaging in self-care activities?						
Internal factors	3	14%	10	59%	7	35%
External factors	6	29%	5	29%	5	25%
Time	10	48%	9	53%	5	0.25

Note. Though the overall sample was 60 participants, 58 participants responded to the qualitative questions. * = Mental Health Workers, in this case, refers to all participants who identified as mental health workers who were not clinical psychologists or affiliated with a university. Examples include therapist, substance use counselor, clinician, mental health nurse, etc.

**Table 6:** Descriptive Statistics for Quantitative Measures.

Measure	*n*	Minimum	Median	Maximum	Mode	Mean	SD
MSPSS							
Total	55	1.75	5.87	7	7	5.8	1.00
Significant Others	55	1.75	6.63	7	7	6.07	1.16
Family	55	1	5.5	7	5.5	5.46	1.29
Friends	55	1.38	5.88	7	7	5.88	1.11
GAD-7	55	7	10	28	10	12.4	4.9
PHQ-9	55	9	13	36	10	14.05	5.7

Note. Though the overall sample was 60 participants, the data included in these analyses was sourced from complete participant responses to the measures (*n* = 55). Incomplete participant responses (*n* = 5) to the MSPSS, GAD-7, and PHQ-9 were not included.

## Data Availability

Data used in this publication are available upon request.
